# Supplementary material for: Polarization-state-resolved high-harmonic spectroscopy of solids
Source: Nat Commun. 2019 Mar 21;10:1319. doi: 10.1038/s41467-019-09328-1 (PMC6428929; doi:10.1038/s41467-019-09328-1)
Supplement: Supplementary file 1 — Supplementary Information [file 41467_2019_9328_MOESM1_ESM.pdf]

Supplementary Information for

Polarization-state-resolved high-harmonic spectroscopy of solids

N. Klemke, N. Tancogne-Dejean, G. M. Rossi, Y. Yang, F. Scheiba,

R. E. Mainz, G. Di Sciacca, A. Rubio, F. X. Kärtner, and O. D. Mücke

(Dated: February 20, 2019)

Supplementary Note 1: Experimental high-harmonic generation setup

Supplementary Figure 1 shows the experimental setup used for high-order harmonic generation (HHG) from crystalline solids. For a detailed discussion of this setup, see Methods section.

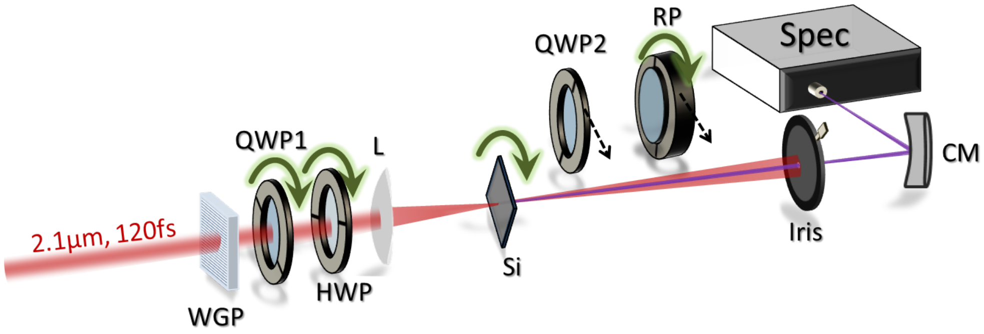

Supplementary Figure 1. **Experimental HHG setup.** WGP, Wire-grid polarizer; QWP1, quarter-wave plate; HWP, half-wave plate; L, CaF_2 lens, $f = 25\,\text{cm}$; Si, (100)-cut 2-µm-thin silicon; RP, Rochon polarizer; QWP2, tunable quarter-wave plate; CM, curved mirror; SPEC, UV-NIR spectrometer.

Supplementary Note 2: Precision of ellipticity and major-axis angle calibration

When measuring the harmonic response maps as function of driver-pulse ellipticity ϵ and sample rotation θ, we require that the major axis of the driving field polarization ellipse

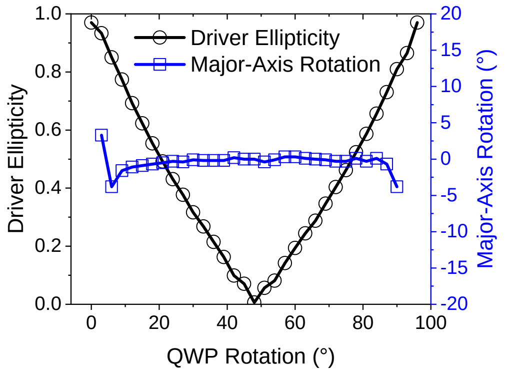

Supplementary Figure 2. **Ellipticity and major-axis angle calibration.** The ellipticity and major-axis orientation of the driving field as measured with a polarizer and powermeter. The major axis is kept constant to $\pm 2°$ within $|\epsilon| < 0.8$ and $\pm 4°$ for $|\epsilon| > 0.8$.

remains constant ideally over the entire range of ϵ values. Furthermore, also the precise value of ϵ is important. As can be seen from the calibration data shown in Supplementary Figure 2, the combination of a quarter-wave plate (QWP) and a half-wave plate (HWP) allows us to vary to ellipticity ϵ within [0, 0.98], while the major axis of the polarization ellipse remains constant to $\pm 2°$ within $|\epsilon| < 0.8$ and $\pm 4°$ for $|\epsilon| > 0.8$.

Supplementary Note 3: Intensity scaling of the harmonics in silicon

Perturbatively, the nth-harmonic intensity, I_n, scales as $I_n \sim I^n$. Here, I is the driving pulse intensity. Supplementary Figure 3 shows the intensities of HH5-HH9 from Si when varying the driving intensity. For intensities above 0.3 TW cm^{-2}, all harmonics clearly deviate from the perturbative power law. All our experiments and simulations are performed above this intensity in the nonperturbative regime.

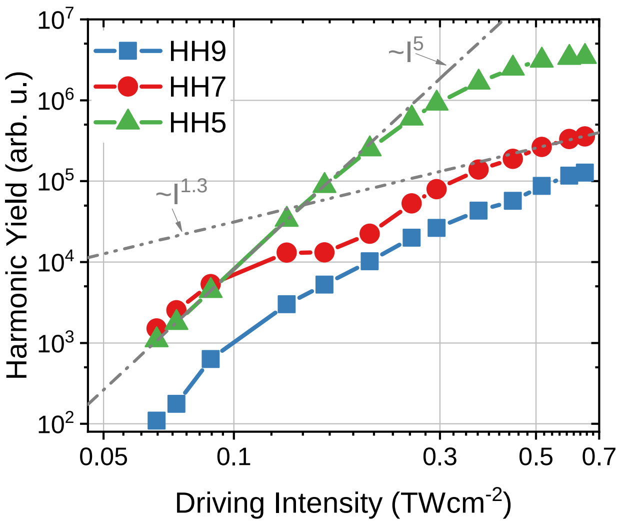

Supplementary Figure 3. **Dependence of the measured harmonic yield on the driving intensity for $\epsilon = 0$.**

Supplementary Note 4: Laue X-ray diffraction of silicon samples

In our experiments, we mainly use free-standing 2-µm-thin (100)-cut Si samples (by Norcada). To verify the monocrystallinity and to determine and align the exact crystal orientation, in-house Laue X-ray diffraction is performed employing a Mo anode X-ray tube on these samples. Supplementary Figure 4 shows a typical diffraction pattern.

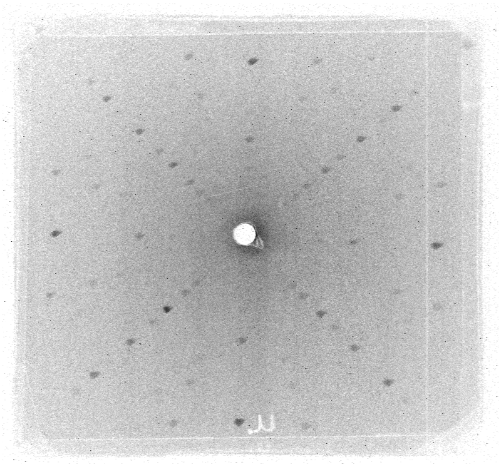

Supplementary Figure 4. **Laue X-ray diffraction pattern from a (100)-cut Si sample.**

Supplementary Note 5: Comparison of time-dependent density-functional theory (TDDFT) computed and experimentally measured HHG spectra

Supplementary Figure 5 shows a comparison of a computed HHG spectrum to a corresponding experimental spectrum. Note that in our experiments we only detect harmonics up to

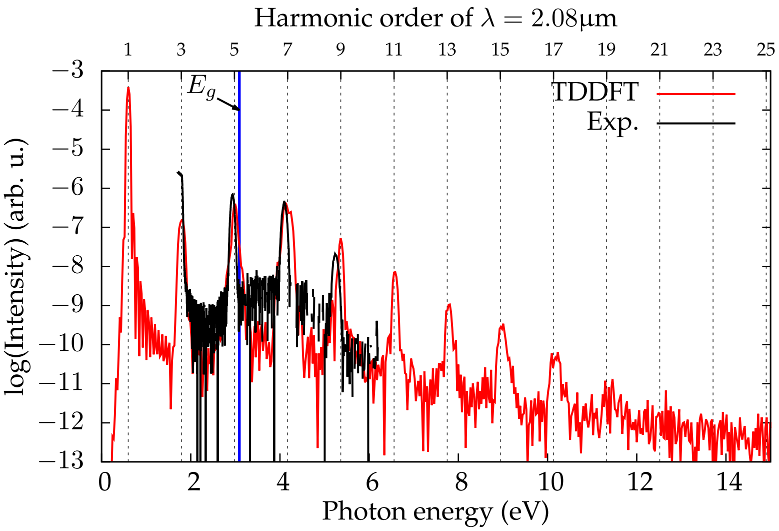

Supplementary Figure 5. **Computed harmonic spectrum of Si compared to the experimental spectrum.** The driving intensity in vacuum is 6×10^{11} W cm^{-2}, driving wavelength is $\lambda = 2.08$ μm. The driving field is linearly polarized and the polarization direction is taken along the ΓX direction. The blue line indicates the theoretical band-gap calculation result using the TB09 MGGA functional. The experimental wavelength is 2.1 μm.

HH9 due to the spectrometer used (Ocean Optics UV-VIS HR4000). Our TDDFT calculations, described in the Methods section, predict that harmonics up to HH19 in the XUV spectral region are generated for our experimental conditions.

Note that due to the shorter driver wavelength and longer driver pulse duration, no noisy structure originating from the interference between interband and intraband emission channels appears in our TDDFT computed HHG spectrum, i.e., we obtain clean harmonic peaks. This is totally consistent with the interpretation of the different HHG mechanisms and the wavelength-scaling plot Fig. 3 in our previous work[1].

Supplementary Note 6: Joint density of states (JDOS)

The generation of harmonic photons by the interband mechanism corresponds to the recombination of an excited electron in a conduction band with a hole in a valence band. Therefore, the possibility of emitting a photon by the interband mechanism at a given energy is directly linked to the density of optical transitions available at this photon energy[1]. This quantity is also known as the joint density of states (JDOS). Due to the nonperturbative nature of the process of high-harmonic generation, carriers intially created by interband transitions near the Γ point in Si, start to explore a region of the BZ around the minimal band gap. In order to estimate the region of the BZ seen by the driven electrons, one can resort to the so-called "acceleration theorem", which gives an upper limit to this region. In fact, the scattering between electrons, as well as the coupling to other bands (interband transitions) reduces the exploration of the electron wavepacket[2]. The JDOS is given by

$$\mathrm{JDOS}(\omega) \propto \sum_{\mathrm{v,c}} {\sum_{\mathbf{k}}}' \delta(E_{\mathrm{c},\mathbf{k}} - E_{\mathrm{v},\mathbf{k}} - \omega)\,, \tag{1}$$

where the indices v,c denote valence and conduction bands, ${\sum_{\mathbf{k}}}'$ denotes a sum over the explored region of the BZ determined by the acceleration theorem in the case of independent electrons, i.e., $\mathbf{k}(t) = -\frac{1}{c}\mathbf{A}(t)$. More precisely, from the knowledge of the time-dependent vector potential $A(t)$, one obtains the region in $\mathbf{k}$-space that can be explored by the electrons during the pulse duration. For each set of laser parameters, we thus obtain a different set of $\mathbf{k}$-points, that we later use to evaluate Eq. (1).

In Supplementary Figure 6, we show the evolution of the JDOS with the ellipticity of the driving field and sample rotation. We found that for all sample rotations and ellipticities,

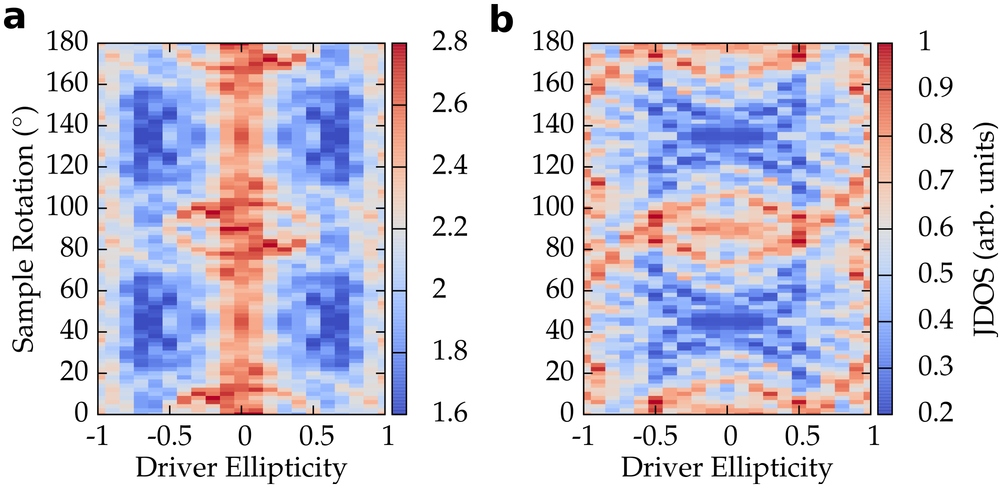

Supplementary Figure 6. **Dependence of the JDOS on driver ellipticity and sample rotation.** JDOS at the energy of **(a)** HH7 and **(b)** HH9. HH5 is not shown as it lies below the experimental band gap of Si. Calculations are performed for an intensity in vacuum of 0.6 TW cm^{-2}.

the JDOS at the energy of HH7 is much higher than at the energy of HH9, indicating that interband transitions are stronger for HH7 compared to HH9.

Supplementary Note 7: Calculation of the center of mass

The center of mass (COM) curves displayed in Fig. 1a-c are calculated using

$$\mathrm{COM}(\theta) = \frac{\sum\limits_{|\epsilon|<0.5} \epsilon \cdot I_{\mathrm{HH}}(\epsilon, \theta)}{\sum\limits_{|\epsilon|<0.5} I_{\mathrm{HH}}(\epsilon, \theta)}, \tag{2}$$

where I_{HH} is the yield of the respective harmonic order, and ϵ, θ are again driver ellipticity and sample rotation. The centers of mass are calculated in an interval $|\epsilon| < 0.5$ to emphasize the asymmetric response in the most intense region of the ellipticity profiles. Supplementary Figure 7 shows the COM curves of HH5-HH9 in Si as shown in Fig. 1a-c in the main text. Note that COM = 0 is required by mirror symmetry along the symmetry axes ΓX and ΓK and that this is well reproduced in our data.

Supplementary Note 8: Circular HH5 from elliptical driver pulses

Supplementary Figure 8 shows a polarizer scan of HH5-HH9 with $\epsilon = 0.4$ and $\theta = \Gamma\mathrm{X} + 5°$. In this case, HH5 has nearly circular polarization with a harmonic ellipticity of $\epsilon_{\mathrm{HH}} = 0.9$.

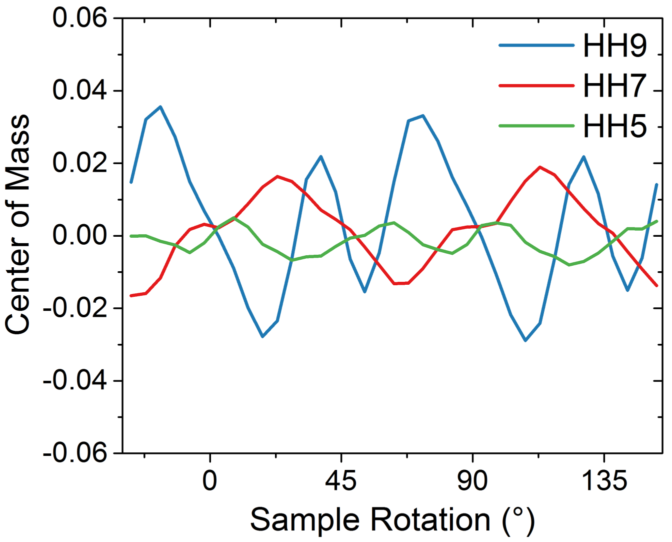

Supplementary Figure 7. **Center of mass (COM) in ϵ-coordinate for HH5, HH7 and HH9 from Si as shown in Fig. 1a-c in the main text.**

HH7 and HH9 are elliptically and linearly polarized, respectively. The driving laser major axis is aligned at 0°. Note that the harmonics' major axes are rotated with respect to each other and HH7-HH9 are nearly perpendicular to the driving ellipse.

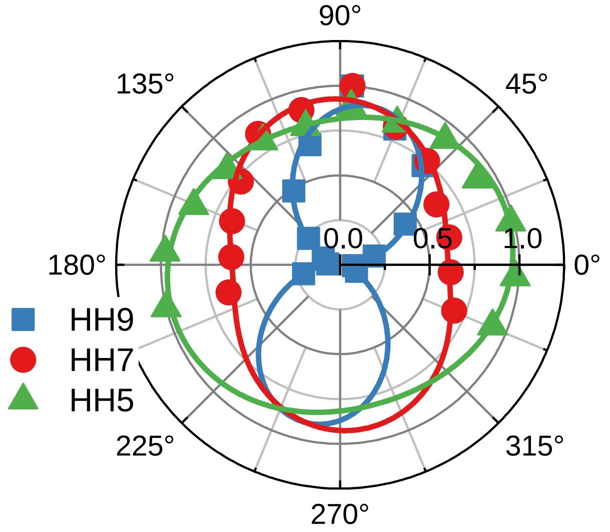

Supplementary Figure 8. **Circularly polarized HH5 from an elliptically polarized driver.** Polarizer scan of HH5-HH9 for $\epsilon = 0.4$ and $\theta = \Gamma X + 5°$.

Supplementary Note 9: Stokes polarization parameters of harmonics from silicon

The polarization state of light can be fully characterized by the four Stokes parameters S_0, S_1, S_2 and S_3 [3,4] defined via

$$S_0 = |E_x|^2 + |E_y|^2 \tag{3}$$

$$S_1 = |E_x|^2 - |E_y|^2 \tag{4}$$

$$S_2 = 2\,\mathrm{Re}(E_x E_y^*) \tag{5}$$

$$S_3 = -2\,\mathrm{Im}(E_x E_y^*)\,. \tag{6}$$

E_x and E_y denote the electric field components along the x- and y-directions. The degree of polarization (DOP) is defined as

$$\mathrm{DOP} = \frac{\sqrt{S_1^2 + S_2^2 + S_3^2}}{S_0}, \quad \mathrm{DOP} \in [0,1]\,. \tag{7}$$

DOP = 1 for completely polarized light, and DOP = 0 for completely unpolarized light.

If $I(\phi_{\mathrm{pol}}, \delta_{xy})$ denotes the transmitted intensity after a polarizer with angle ϕ_{pol} and an introduced phase shift δ_{xy} between E_x and E_y, then the Stokes parameters can be determined from

$$S_0 = I(0,0) + I(\pi/2, 0) \tag{8}$$

$$S_1 = I(0,0) - I(\pi/2, 0) \tag{9}$$

$$S_2 = 2I(\pi/4, 0) - S_0 \tag{10}$$

$$S_3 = S_0 - 2I(\pi/4, \pi/2)\,. \tag{11}$$

Hence, S_0, S_1 and S_2 can be measured with a polarizer only. For measuring S_3, an additional QWP is required to introduce $\delta_{xy} = \pi/2$. The normalized Stokes parameters (S_1/S_0, S_2/S_0, S_3/S_0) can be measured throughout the IR to XUV regions[5,6].

We measured the Stokes parameters of the harmonics from Si in two experiments: for circular driver pulses versus sample rotation (see Supplementary Figure 9), and for fixed sample orientation along $\theta = \Gamma\mathrm{X} + 5^\circ$ as function of driver ellipticity ϵ (Supplementary

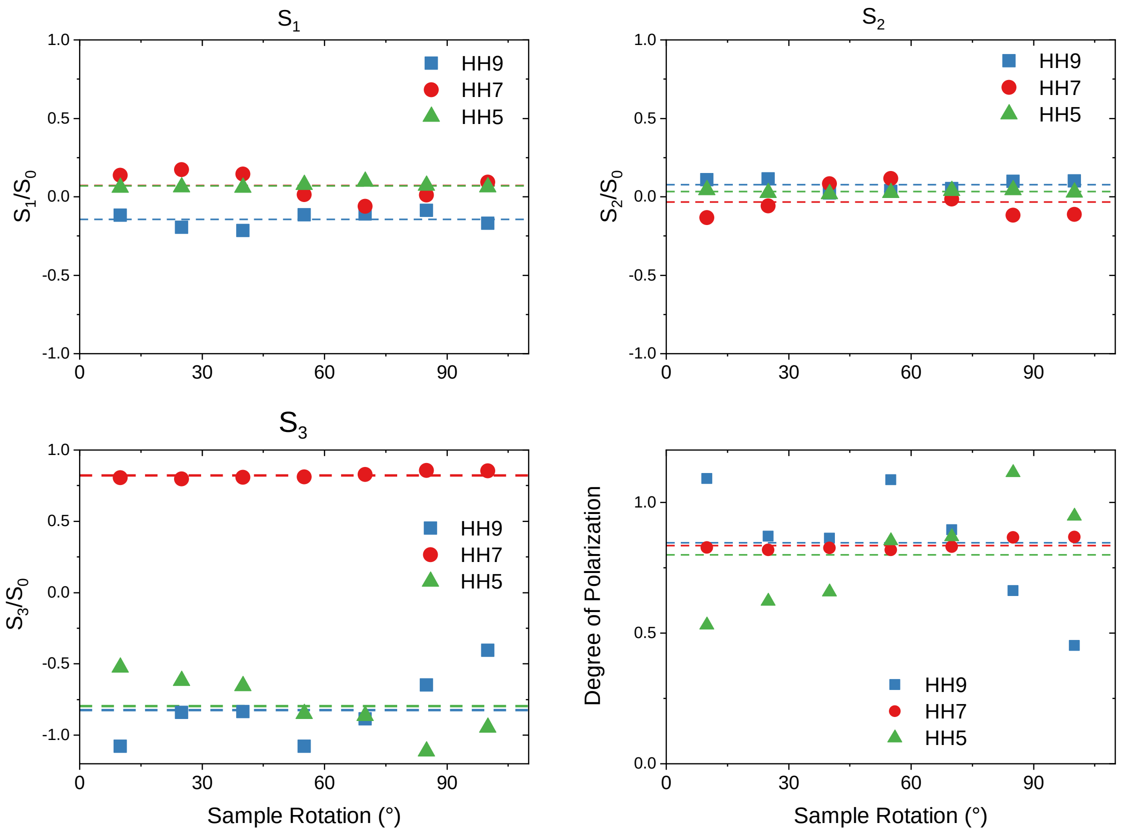

Supplementary Figure 9. **Stokes parameters and degree of polarization versus sample rotation for circular driver polarization.** The dashed lines indicate the mean values.

Figure 10). Note that the measurement of S_3 is known to be prone to experimental errors[4] due to the insertion of a QWP and comparison with another independent measurement without the QWP. Our tunable QWP (from Alphalas) itself might absorb or reflect light and might also not introduce a precise $\pi/4$ phase shift for the different harmonics. We believe that this is the reason for the scatter of S_3 values in Supplementary Figure 9. However, as stated in the main text and as can be seen from Supplementary Figure 2, experimentally a circular polarization was only realized to $\epsilon = 0.98$, therefore small variations of S_3 might also be caused by a minor deviation from perfectly circular driver polarization.

The different signs of S_3 of the individual harmonics observed in Supplementary Figure 9 are another proof of circular harmonics with alternating helicities. S_1 and S_2 are near zero, as one would expect for circular harmonics. The DOP values, that are comparable to reported values for the generation of circular harmonics from atomic and molecular gases[7,8], show that the harmonics are highly polarized.

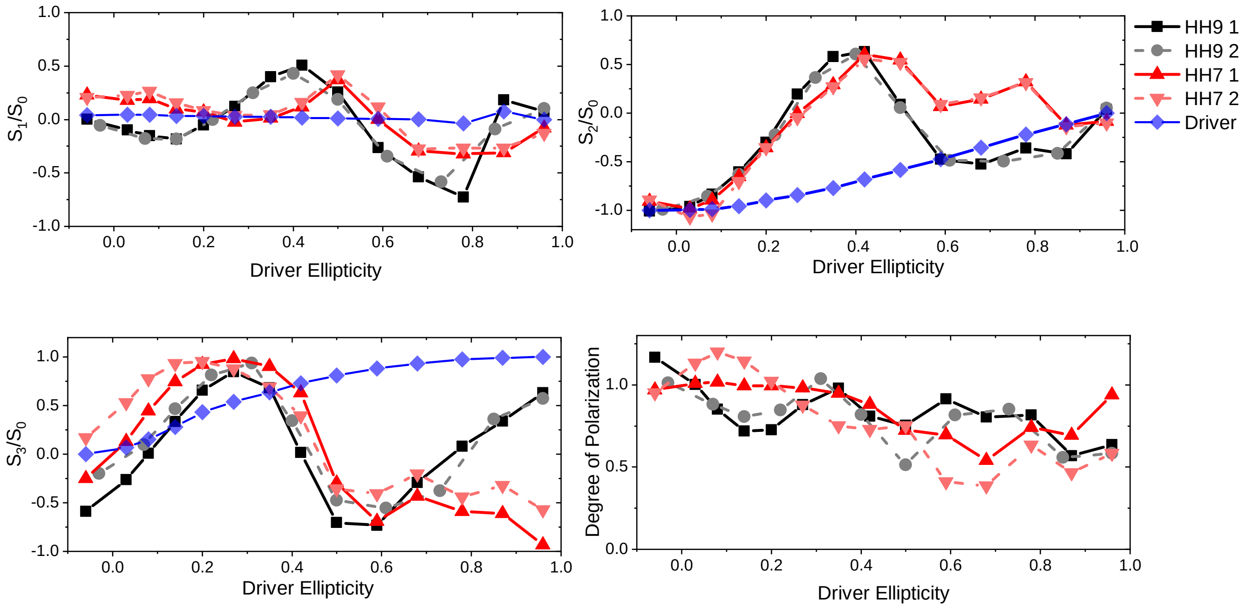

Supplementary Figure 10. **Stokes parameters and degree of polarization versus driver ellipticity ϵ for fixed sample orientation $\theta = \Gamma X - 5°$.** Two independent measurements denoted by 1 and 2 are shown.

Supplementary Figure 10 shows the evolution of the Stokes parameters of HH7 and HH9 when varying the driver-pulse ellipticity ϵ, while keeping the major-axis orientation fixed. In Supplementary Figure 10, also the measured Stokes parameters of the driving field are displayed. In order to also estimate the experimental uncertainty, we repeated the same measurements twice. One can see clear deviations from the driver in the polarization-response of the various harmonics in all Stokes parameters S_i. For S_1 and S_2, both measurements yield almost the same values. For S_3 and DOP, the insertion of the QWP introduces some uncertainty that, however, does not change the qualitative behavior.

These time-integrated measurements unambiguously rule out the possibility that what we measure to be circular harmonics are in fact elliptically polarized harmonics with a major axis rotating in time or that the harmonics' ellipticity changes in time. In these cases, we would still measure a harmonics' intensity that would be constant over polarizer rotation after the second QWP. This is certainly not the case, as Supplementary Figure 11 shows for the two cases of circular HH7 and HH9 from elliptical driver at $\theta = \Gamma X - 5°$.

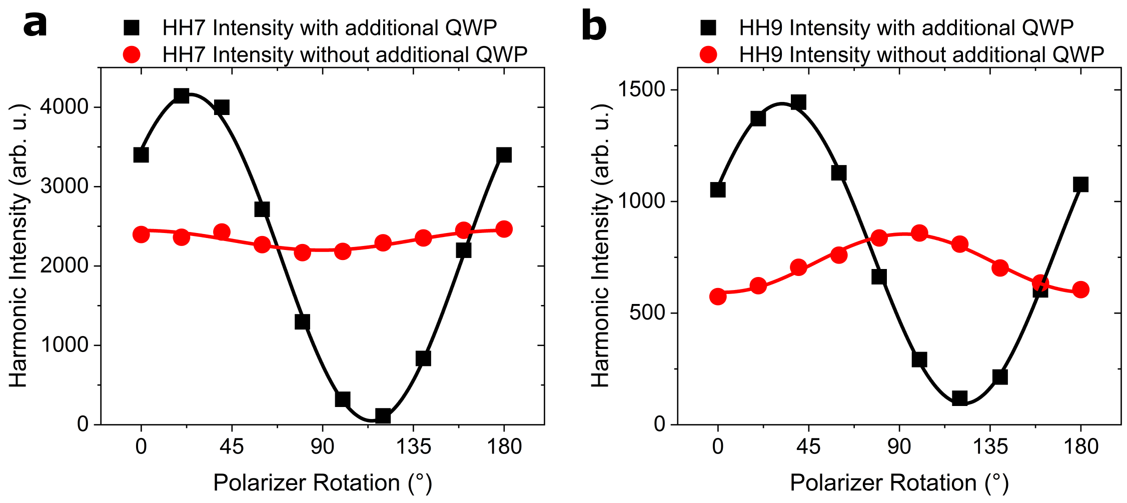

Supplementary Figure 11. **Conversion to linear polarization with a second quarter-wave plate.** Circular harmonics are converted to linear with a second QWP for driving conditions of $\epsilon = 0.3$ (**a**) and $\epsilon = 0.6$ (**b**). Sample rotation is $\theta = \Gamma\mathrm{X} - 5°$. Solid lines are $\sin^2$-fits.

Supplementary Note 10: Time-domain characterization of circular harmonic from silicon

In order to temporally characterize harmonic pulses generated in the nonperturbative interaction regime and thus to confirm a time evolution similar to the one theoretically predicted in Fig. 4 in the main text, we have performed two-dimensional spectral shearing interferometry[9–11] (2DSI) on HH3 from silicon. 2DSI measures the spectral phase of a pulse by making use of two strongly chirped or narrowband filtered pulses (so-called ancillae pulses), that are a replica of each other with a slight shear in central frequency, that need to be temporally synchronized with the pulse under test. The sum frequencies of the narrowband ancillae pulses with the pulse under test lead to an interference pattern in the upconverted wavelength range that encodes the group delay. We have used a portion of the pump pulses of our OPA (800 nm, 150 fs) as ancillae pulses and focused them together with the HHG beam onto a 20-µm-thin BBO crystal (type II, $\theta = 44°$) for sum-frequency generation (SFG). We collected the upconverted light and sent it to an Ocean Optics HR4000 spectrometer. Contrary to the other measurements in this work, here, our 2.1 µm driving pulses had a slightly shorter pulse duration of 80 fs.

Since the type-II BBO only phase matches perpendicular polarization components, the detected SFG for a given 800 nm polarization is always generated with the perpendicular

polarization component of the HHG probe pulse. In order to measure both polarization components E_x and E_y of circular HH3, we rotated the BBO crystal by 90° and, correspondingly, the polarization of the 800 nm ancillae pulses with a HWP. Thus we measure E_x and E_y independently. To reconstruct the spatiotemporal profile of the pulse from the two measurements, one needs to determine the relative amplitudes and the relative phase δ between the two components E_x and E_y. Both have been determined in a separate measurement of the polarization ellipse of the harmonic, as shown in Supplementary Figure 1. The relative phase is[3]

$$\delta = \arccos\left(\frac{\tan(2\psi)\cdot(E_x^2 - E_y^2)}{2E_xE_y}\right). \tag{12}$$

Here, ψ is the major-axis angle of the polarization ellipse relative to the x-component. We determined δ for an instantaneous frequency ∼20 fs before the center of the pulse, at which time both x- and y-components exhibit the same instantaneous frequency. We then delayed the reconstructed field $E_y(t)$ with respect to $E_x(t)$ by the time that corresponds to the relative phase of that frequency. The $\lambda/2$-delay that is introduced by the rotation of the HWP in the ancillae beam introduces an experimental uncertainty in determining the arrival times by one optical period, that, for our relatively long pulses, has no significant effect on the pulse envelope shape. Supplementary Figure 12 shows the measured spatiotemporal electric field of HH3 with a circular driver and its projections onto the corresponding planes.

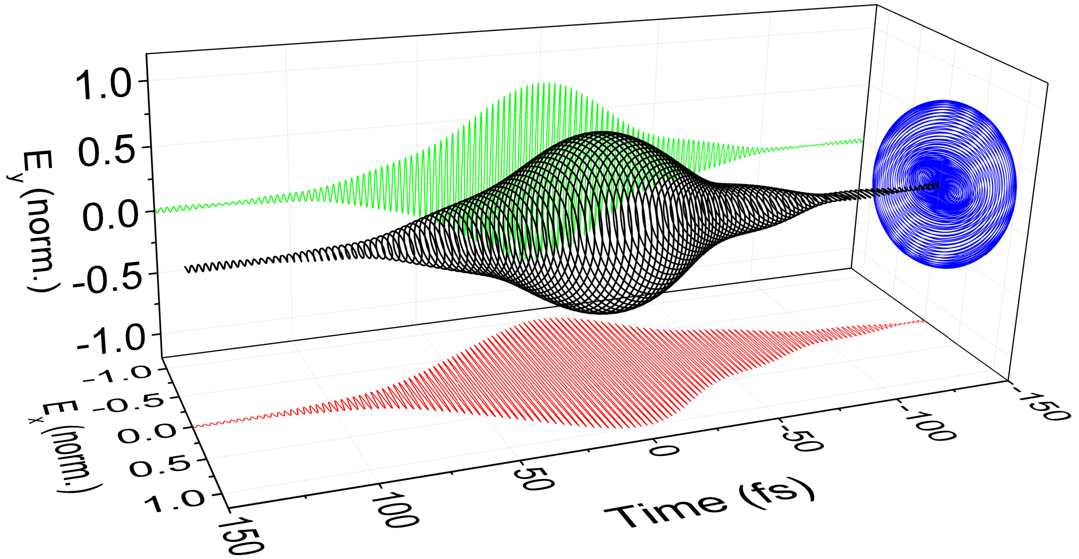

Supplementary Figure 12. **Measured spatiotemporal electric field of HH3 from silicon with a circular driver.**

Similar to other pulse characterization techniques (such as FROG or SPIDER), the CEP of the harmonic can not be derived from the 2DSI measurement and is set to 0 in this case. The determination of the relative phase from an independent polarization scan directly behind the sample ensures that the reconstructed field is unaffected by parasitic influences on δ, e.g., due to reflection phase shifts between s- and p-components on metallic mirrors while sending the harmonic to the 2DSI setup.

The FWHM pulse duration of the x- and y-components are 61 fs and 50 fs, respectively. From conventional nonlinear optics one would expect an $n^{-1/2}$-dependence of the pulse duration of the nth harmonic. In our case $80\,\text{fs}/\sqrt{3} = 46\,\text{fs}$, which is reasonably close to our measured values within experimental precision.

Supplementary Note 11: Coherence of higher harmonics from silicon

As discussed in [12,13,14], coherence comprises the concepts of transverse ('spatial') coherence and longitudinal ('temporal') coherence. A standard test of transverse coherence is the well-known Young's double-slit diffraction technique, which is routinely used (often employing a pinhole pair) to demonstrate spatial coherence of HHG from gases[15,16]. A standard test of temporal coherence is to perform Michelson interferometry[12,13,17,18].

We have set up a balanced Michelson interferometer consisting of a 50/50-coated UV fused silica (UVFS) beam splitter and two mirrors, one of which was mounted on a translation stage (PI M-110) to vary the optical path length of this arm with respect to the other (see Supplementary Figure 13a). The extra disperson that is added in one arm due to threefold propagation through the UVFS substrate was compensated by an additional UVFS plate in the other arm. Both beams were overlapped in space at the output of the interferometer and coupled into the spectrometer. The spectrally-resolved high-harmonic peaks then show interference fringes separated by

$$\Delta\nu = \frac{1}{\Delta t} = \frac{2c}{L}, \tag{13}$$

with $\Delta\nu$ the fringe separation in the frequency domain, Δt the optical delay time, c the speed of light, and L the path length difference between the two arms. Note that the pulse duration in this experiment is 80 fs.

Supplementary Figure 13b depicts the evolution of the spectral fringes for HH5 for $\epsilon = 0$

and two cases, for which HH5 is circularly polarized ($\epsilon = 0.45, \theta = \Gamma X + 5°$ and $\epsilon \approx 1$). One can hardly see a difference in fringe visibility between the different driving conditions, which implies that their coherence properties are very similar. To quantify that, we tracked the oscillating intensity at the center wavelength of the harmonic peak in small time steps and evaluated the fringe visibility as

$$V = \frac{I_{\text{max}} - I_{\text{min}}}{I_{\text{max}} + I_{\text{min}}}, \tag{14}$$

with I_{max} and I_{min} the maximum and minimum intensities of this oscillation, respectively. The fringe visibility is directly connected to the degree of coherence[14]: $|\gamma(\tau)| = V$. V reaches

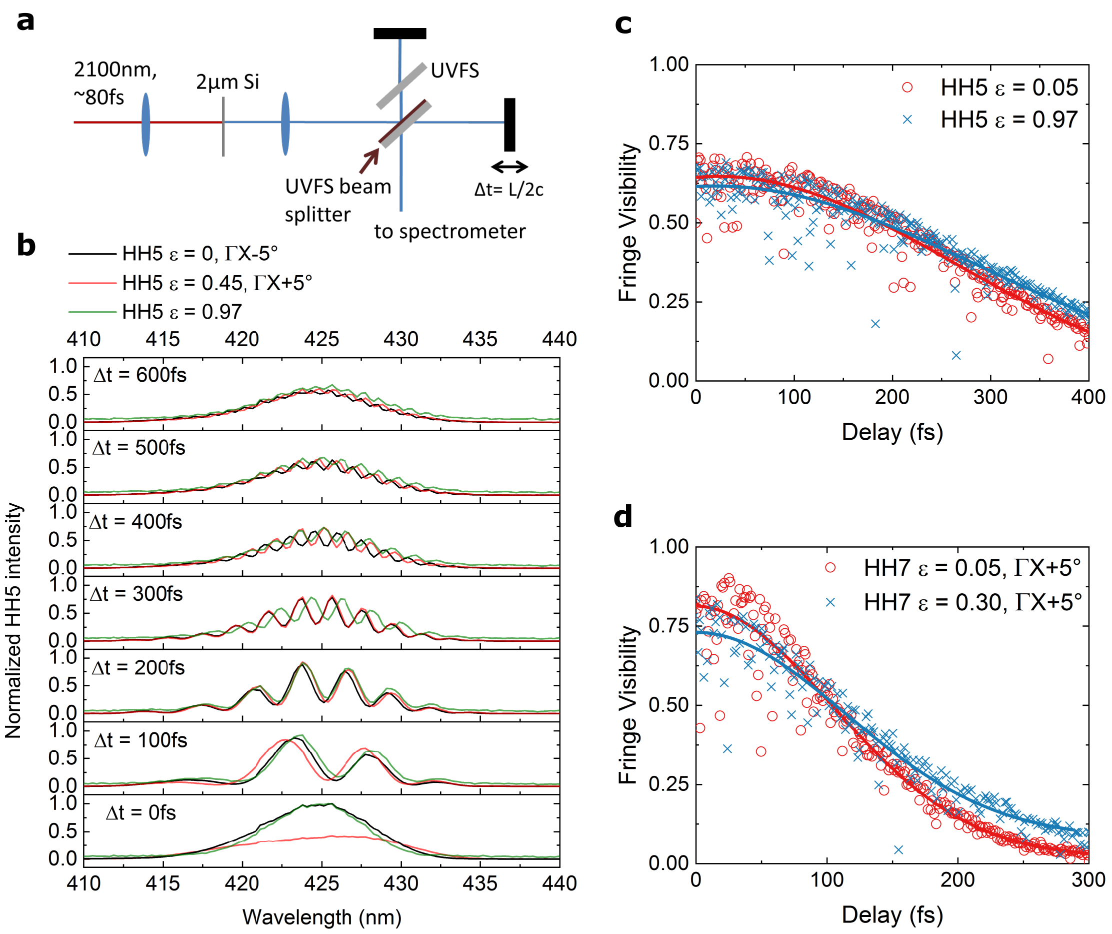

Supplementary Figure 13. **Temporal coherence characterized by Michelson interferometry.** **a** Experimental setup. UVFS, UV fused silica. **b** Spectral fringes of HH5 for different time delays of the interferometer. $\epsilon = 0$ shows the case for linear driver and harmonic polarization, while the two non-zero driver ellipticities generate a circular HH5. In **c-d**, the fringe visibility of HH5 and HH7 at their center wavelength is plotted over time, each panel showing one case for linear harmonic and one for circular harmonic. The solid lines indicate Gaussian fits.

the highest value for very small delays and gradually decreases in a Gaussian manner, as can be expected for Gaussian ultrashort pulses[19]. We define the coherence time τ_c as the time after which $V < 0.5$. For both cases of HH5 in Supplementary Figure 13c, τ_c = 187 fs. Supplementary Figure 13d shows V for two cases of HH7, one for linear driver polarization and one for circularly polarized HH7 obtained from elliptical driver pulses. Here, $\tau_c = 105$ fs. For both HH7 and HH5 we find the coherence time to be independent of the harmonic's polarization state (linear or circular). The experimentally observed maximum fringe visibilities are consistent with similar interferometry studies on the coherence of gas-phase HHG[17,19,20].

Note that the fringe visibility data shown in Fig. 13c-d actually represent a lower limit for the fringe visibility and the extracted coherence time: any interferometer imbalance, temporal undersampling of the fringes due to too large delay steps (explaining the scattered data points significantly below the Gaussian fits) or caused by interferometer vibrations, and especially the finite spectral resolution all lead to a systematic reduction of the visibility that might affect the extracted coherence time. These experimental issues become more relevant at higher frequencies corresponding to higher harmonic orders.

We have also confirmed the spatial coherence in a double-slit diffraction experiment for HH5. We have placed two pieces of optical filters (BG18, Schott) in front of the double slit

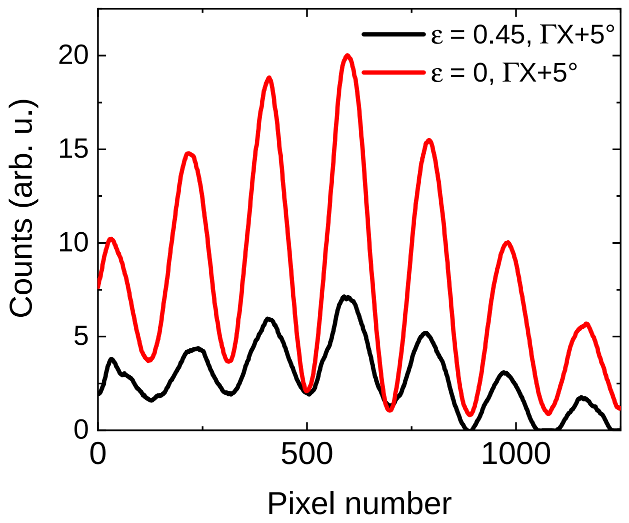

Supplementary Figure 14. **Spatial coherence confirmed by double-slit diffraction pattern.** HH5 after propagation through a double slit with a slit width of 70 μm and separation of 300 μm. The red curve shows a linearly polarized HH5, the black curve a circularly polarized HH5.

to exclusively record HH5 on a CCD camera in the far field behind the double slit. The resulting interference pattern of HH5 for a case of linear polarization and a case of circular polarization are shown in Supplementary Figure 14. We conclude that in both cases, the fifth harmonic is spatially coherent. The reduced fringe visibility for the circular case originates most probably from the lower signal-to-noise ratio in this case.

SUPPLEMENTARY REFERENCES

[1] Tancogne-Dejean, N., Mücke, O. D., Kärtner, F. X. & Rubio, A. Impact of the electronic band structure in high-harmonic generation spectra of solids. *Phys. Rev. Lett.* **118**, 087403 (2017).

[2] Tancogne-Dejean, N., Mücke, O. D., Kärtner, F. X. & Rubio, A. Ellipticity dependence of high-harmonic generation in solids originating from coupled intraband and interband dynamics. *Nature Commun.* **8**, 745 (2017).

[3] Collett, E. *Field Guide to Polarization* (SPIE Press, Bellingham, WA, 2005).

[4] Schaefer, B., Collett, E., Smyth, R., Barrett, D. & Fraher, B. Measuring the stokes polarization parameters. *Am. J. Phys.* **75**, 163 (2007).

[5] Koide, T. *et al.* Elliptical-polarization analyses of synchrotron radiation in the 5-80-eV region with a reflection polarimeter. *Nucl. Instr. and Meth. A* **308**, 635–644 (1991).

[6] Huang, P.-C. *et al.* Polarization control of isolated high-harmonic pulses. *Nature Phot.* **12**, 349–354 (2018).

[7] Kfir, O. *et al.* Generation of bright phase-matched circularly polarized extreme ultraviolet high harmonics. *Nature Phot.* **9**, 99–105 (2014).

[8] Veyrinas, K. *et al.* Molecular frame photoemission by a comb of elliptical high-order harmonics: a sensitive probe of both photodynamics and harmonic complete polarization state. *Faraday Discuss.* **194**, 161–183 (2016).

[9] Birge, J. R., Ell, R. & Kärtner, F. X. Two-dimensional spectral shearing interferometry for few-cycle pulse characterization. *Opt. Lett.* **31**, 2063–2065 (2006).

[10] Birge, J. R., Crespo, H. M. & Kärtner, F. X. Theory and design of two-dimensional spectral shearing interferometry for few-cycle pulse measurement. *J. Opt. Soc. Am. B* **31**, 1165–1173 (2010).

[11] Chia, S.-H., Scheiba, F., Rossi, G. M., Mücke, O. D. & Kärtner, F. X. CLEO-EU 2017, paper CF-13.3, Munich, Germany, June 25-29, 2017.

[12] Attwood, D. & Sakdinawat, A. *X-Rays and Extreme Ultraviolet Radiation: Principles and Applications* (Cambridge University Press, 2016), second edn.

[13] Mandel, L. & Wolf, E. *Optical Coherence and Quantum Optics* (Cambridge University Press, 1995), second edn.

[14] Hecht, E. *Optics* (Pearson, 2016), fifth edn.

[15] Bartels, R. A. *et al.* Generation of spatially coherent light at extreme ultraviolet wavelengths. *Science* **297**, 376–378 (2002).

[16] Popmintchev, T. *et al.* Bright coherent ultrahigh harmonics in the keV X-ray regime from mid-infrared femtosecond lasers. *Science* **336**, 1287–1291 (2012).

[17] Bellini, M. *et al.* Temporal coherence of ultrashort high-order harmonic pulses. *Phys. Rev. Lett.* **81**, 297–300 (1998).

[18] Bellini, M. & Hänsch, T. W. Phase-locked white-light continuum pulses: toward a universal optical frequency-comb synthesizer. *Opt. Lett.* **25**, 1049–1052 (2000).

[19] Nabekawa, Y., Shimizu, T., Furukawa, Y., Takahashi, E. J. & Midorikawa, K. Interferometry of attosecond pulse trains in the extreme ultraviolet wavelength region. *Phys. Rev. Lett.* **102**, 213904 (2009).

[20] Kovačev, M. *et al.* Extreme ultraviolet Fourier-transform spectroscopy with high order harmonics. *Phys. Rev. Lett.* **95**, 223903 (2005).
